# The upregulated expression of RFC4 and GMPS mediated by DNA copy number alteration is associated with the early diagnosis and immune escape of ESCC based on a bioinformatic analysis

**DOI:** 10.18632/aging.203520

**Published:** 2021-09-14

**Authors:** Jing Wang, Fei-Fei Luo, Tie-Jun Huang, Yan Mei, Li-Xia Peng, Chao-Nan Qian, Bi-Jun Huang

**Affiliations:** 1 Department of Experimental Research, State Key Laboratory of Oncology in South China, Collaborative Innovation Center for Cancer Medicine, Sun Yat-Sen University Cancer Center, Guangzhou 510060, People’s Republic of China; 2 Department of Nasopharyngeal Carcinoma, Sun Yat-Sen University Cancer Center, Guangzhou 510060, People’s Republic of China; 3 Department of Nuclear Medicine, The Second People’s Hospital of Shenzhen, Shenzhen 518037, People’s Republic of China

**Keywords:** ESCC, RFC4 and GMPS, DNA copy number, early diagnosis, tumor-infiltrating immune cells

## Abstract

Esophageal squamous cell carcinoma (ESCC) is a malignant tumor that commonly occurs worldwide. Usually, Asia, especially China, has a high incidence of esophageal cancer. ESCC often has a poor outcome because of a late diagnosis and lack of effective treatments.

To build foundations for the early diagnosis and treatment of ESCC, we used the gene expression datasets GSE20347 and GSE17351 from the GEO database and a private dataset to uncover differentially expressed genes (DEGs) and key genes in ESCC. Notably, we found that replication factor C subunit 4 (RFC4) and guanine monophosphate synthase (GMPS) were upregulated but have been rarely studied in ESCC. In particular, to the best of our knowledge, our study is the first to explore GMPS and ESCC. Furthermore, we found that high levels of RFC4 and GMPS expression may result from an increase in DNA copy number alterations. Furthermore, RFC4 and GMPS were both upregulated in the early stage and early nodal metastases of esophageal carcinoma. The expression of RFC4 was strongly correlated with GMPS. In addition, we explored the relationship between RFC4 and GMPS expression and tumor-infiltrating immune cells (TILs) in esophageal carcinoma. The results showed that the levels of RFC4 and GMPS increased with a decrease in some tumor-infiltrating cells. Upregulated RFC4 and GMPS with high TILs indicate a worse prognosis.

In summary, our study shows that RFC4 and GMPS have potential as biomarkers for the early diagnosis of ESCC and may played a crucial role in the process of tumor immunity in ESCC.

## INTRODUCTION

Esophageal carcinoma is a common malignant tumor worldwide [1, 2]. This cancer ranked seventh in cancer incidence and sixth in mortality overall in 2018 [3]. Most cases of esophageal cancer, especially in Eastern Europe and Asia, are squamous cell carcinoma [3–5]. Usually, tobacco and alcohol consumption are major risk factors for esophageal carcinoma [6, 7]. Esophagectomy is the major therapy for locoregional esophageal cancer [8]. Although a multidisciplinary approach is used for esophageal cancer treatment, the prognosis of patients with esophageal carcinoma is still poor [9]. In fact, one of the main reasons is that esophageal cancer is usually diagnosed at a late stage [10]. Researchers also believe that an earlier diagnosis is associated with better outcomes than a late diagnosis [11, 12]. However, the lack of early diagnosis markers remains a great challenge for esophageal carcinoma treatment and prognosis [13].

In recent decades, bioinformatics has become an important component of cancer research [14]. In particular, increasing numbers of public datasets, such as The Gene Expression Omnibus (GEO) database and The Cancer Genome Atlas (TCGA) database, have been established for oncology research. Usually, researchers use these datasets to screen tumor-associated biomarkers and excavate potential genetic targets of cancer [15, 16].

In the present study, we focused our research on ESCC. GSE20347 and GSE17351 from GEO and one of our private datasets were used to identify differentially expressed genes (DEGs) in ESCC. Then, Gene Ontology (GO), Kyoto Encyclopedia of Genes and Genomes (KEGG) and protein-protein interaction (PPI) network analyses were used to identify the relevant functions of the DEGs. Among the most significant DEGs, RFC4 and GMPS were both increased in the early stage and early nodal metastases of esophageal carcinoma. When the levels of RFC4 and GMPS increased, we found a decrease in some tumor-infiltrating cells. Therefore, we hypothesize that RFC4 and GMPS are involved in the early progression of esophageal cancer, and mediate the immune escape of esophageal carcinoma.

In summary, our results reveal that RFC4 and GMPS have potential as early diagnostic markers and new immunotherapy targets for ESCC. To date, our study is the first to systematically explore the functions of RFC4 and GMPS in ESCC. Furthermore, the discovery of RFC4 and GMPS can help us better understand the early diagnosis and treatment of esophageal cancer.

## RESULTS

### Identification of DEGs, PPI network construction and hub gene selection in ESCC

**Figure 1:**
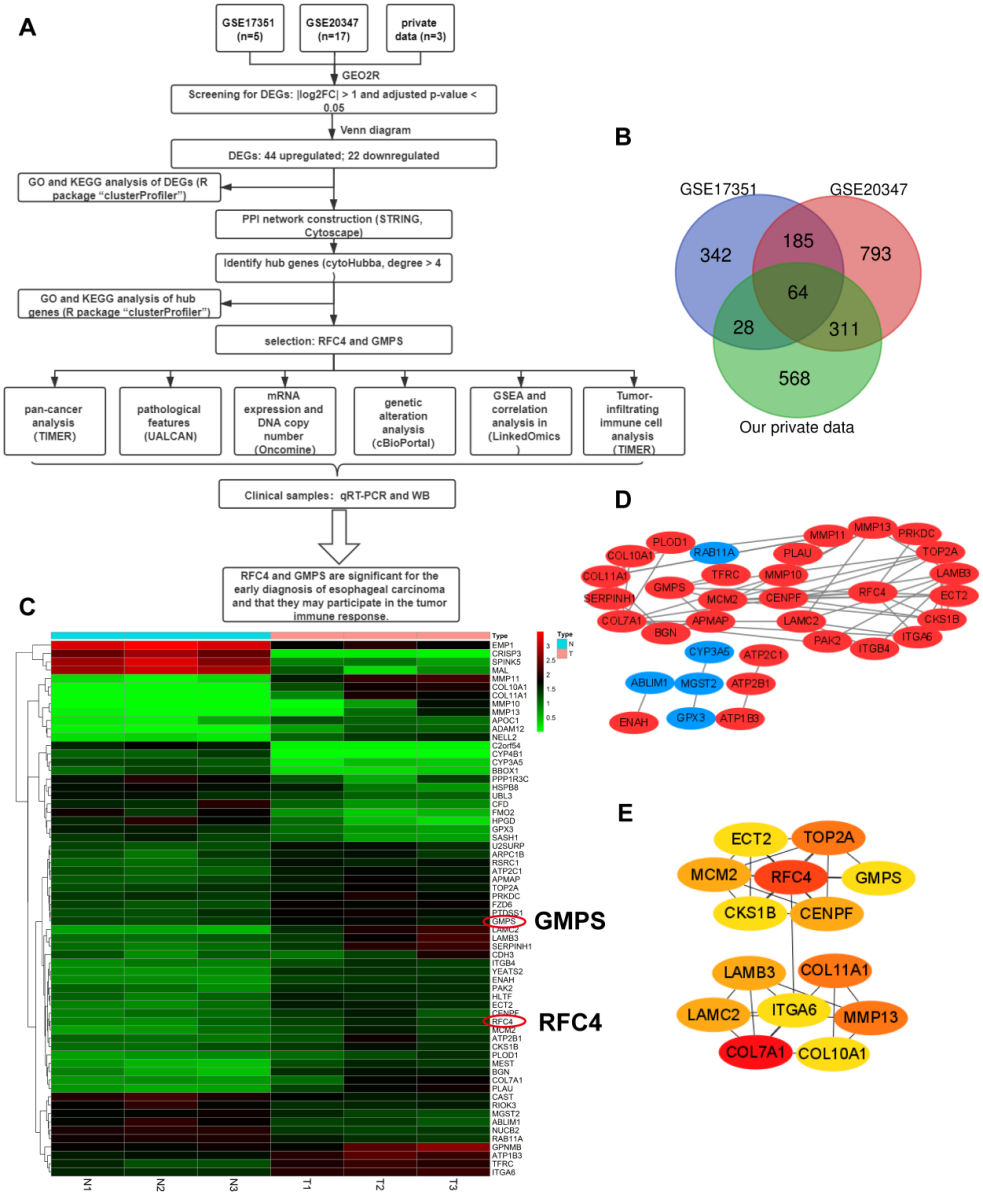
**Differentially expressed genes analyzed in the GSE20347, GSE17351 and private datasets.** (**A**) Flow chart of the data analysis in this study. (**B**) Venn diagrams of the DEGs from the GSE20347, GSE17351 and private datasets. (**C**) Heatmap of 64 DEGs from the private dataset. (**D**) Visual PPI network of 64 DEGs from Cytoscape. Upregulated genes are marked in red; downregulated genes are marked in blue. (**E**) Fourteen hub genes screened by a degree > 4.

Flow chart of the whole data analysis is shown in Figure 1A. According to the comparison of GSE20347, GSE17351 and our private dataset, there were 25 ESCC tissues and 25 normal tissues in the present study. Using GEO2R online tools and Venn diagram software, the results showed that in total, 64 genes were identified as DEGs (Figure 1B, 1C), including 22 downregulated genes and 42 upregulated genes in the ESCC tissues (Supplementary Table 1). Then, we used STRING and Cytoscape tools to construct the PPI network of the DEGs (Figure 1D) and screen the hub genes. The results obtained using the cytoHubba module of Cytoscape showed that 14 genes with a degree >4 were identified as hub genes (Figure 1E), including COL7A1, RFC4, MMP13, COL11A1, TOP2A, LAMB3, LAMC2, CENPF, MCM2, GMPS, CKS1B, ECT2, COL10A1, and ITGA6 (Table 1).

**Table 1:** 14 hub genes.

**Gene symbol**	**Full name**	**Degree score**
COL7A1	Collagen alpha-1(VII) chain	9
RFC4	Replication factor C subunit 4	8
MMP13	Collagenase 3	7
COL11A1	Collagen alpha-1(XI) chain	7
TOP2A	DNA topoisomerase 2-alpha	7
LAMB3	Laminin subunit beta-3	6
LAMC2	Laminin subunit gamma-2	6
CENPF	Centromere protein F	6
MCM2	DNA replication licensing factor MCM2	6
GMPS	guanine monophosphate synthase	5
CKS1B	Cyclin-dependent kinases regulatory subunit 1	5
ECT2	Protein ECT2	5
COL10A1	Collagen alpha-1(X) chain	5
ITGA6	Integrin alpha-6	5

### Functional analysis of DEGs and hub genes

To analyze the potential biological function and signaling pathways of the DEGs and hub genes, GO and KEGG pathway analyses were performed using the R package “clusterProfiler”.

**Figure 2:**
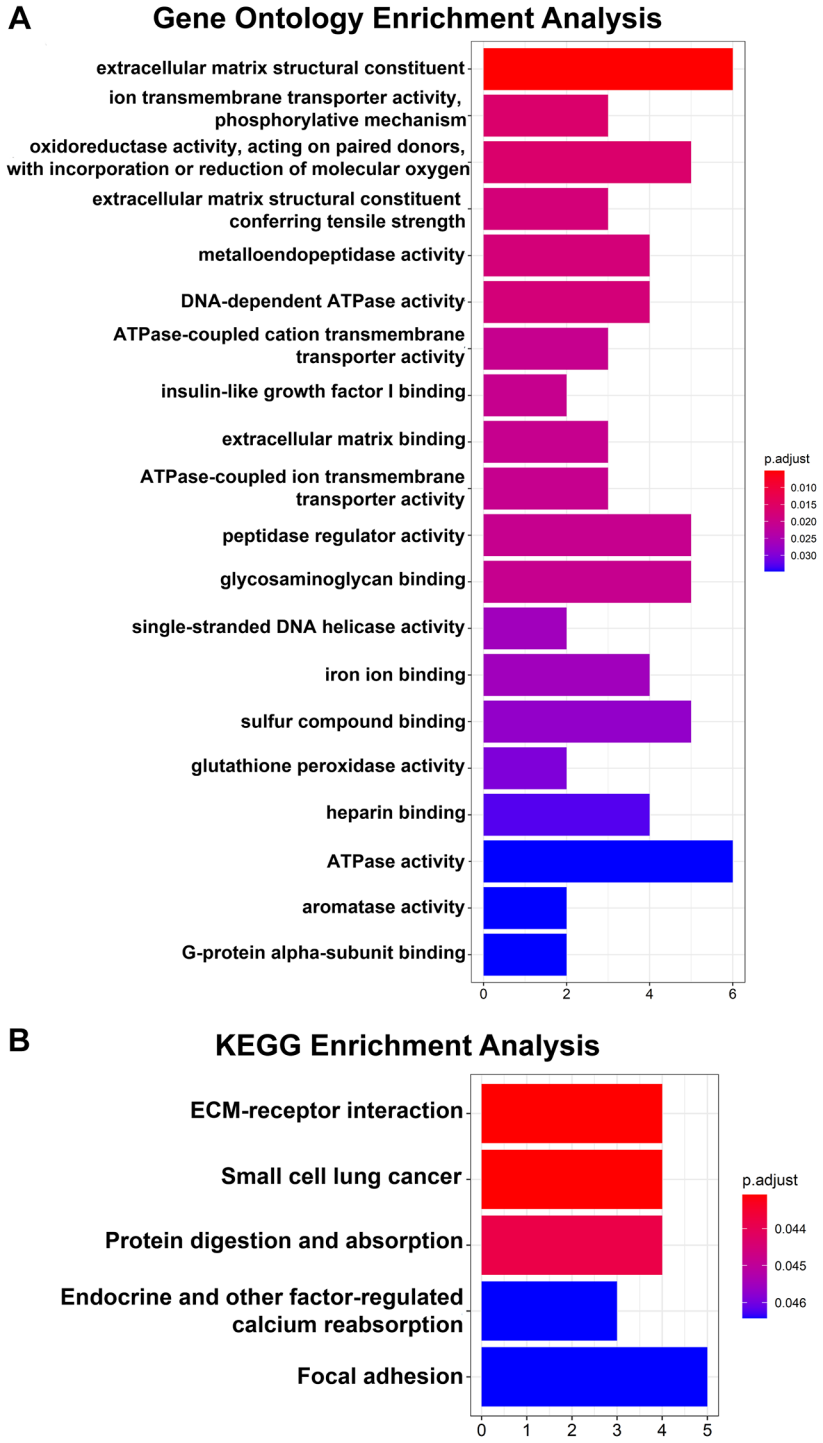
**GO and KEGG pathway enrichment analyses of 64 DEGs.** (**A**) Top 20 GO enrichment analyses of 64 DEGs. (**B**) KEGG pathway analysis of 64 DEGs. *P* < 0.05 indicates statistical significance.

Regarding the DEGs, the GO analysis results showed that the changes in the DEGs were significantly enriched in extracellular matrix structural constituents (Figure 2A). The KEGG pathway analysis revealed that the DEGs were mainly enriched in ECM-receptor interactions (Figure 2B).

**Figure 3:**
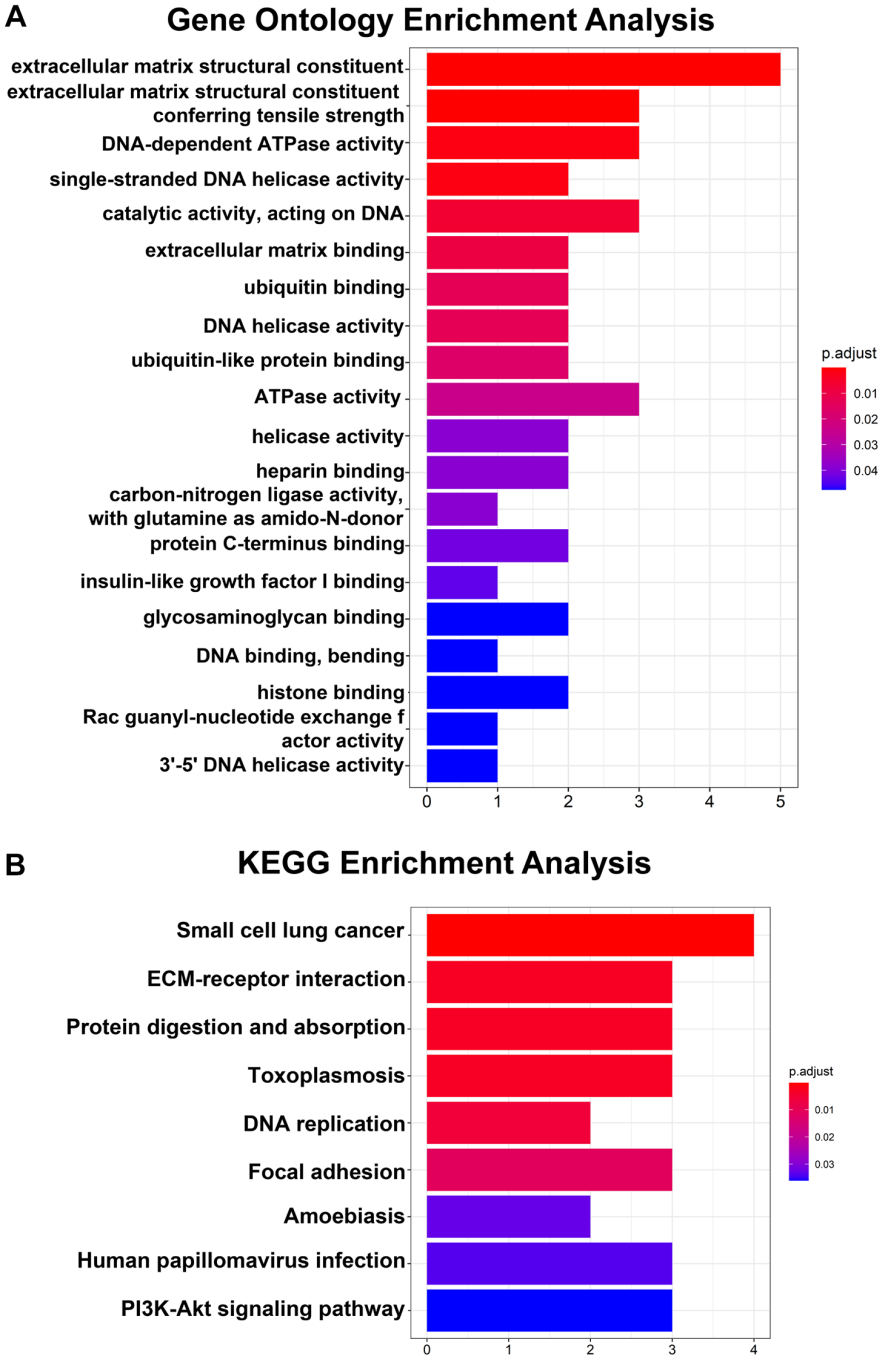
**GO and KEGG pathway analyses of 14 hub genes.** (**A**) Top 20 GO enrichment analyses of 14 hub genes. (**B**) KEGG pathway analysis of 14 hub genes. *P* < 0.05 indicates statistical significance.

Regarding the hub genes, the GO analysis results indicated that the hub genes were mainly enriched in extracellular matrix structural constituents, extracellular matrix structural constituents conferring tensile strength and DNA-dependent ATPase activity and so on (Figure 3A). Moreover, the KEGG pathway analysis revealed that the hub genes were enriched in small cell lung cancer, ECM-receptor interaction and protein digestion and adsorption (Figure 3B).

Meanwhile, by examining the data, we found that RFC4 and GMPS have rarely been reported in ESCC. In particular, to the best of our knowledge, our study is the first to report the relationship between GMPS and ESCC. Therefore, we focused our attention on RFC4 and GMPS in ESCC.

### RFC4 and GMPS were upregulated in cancers, especially ESCC

**Figure 4:**
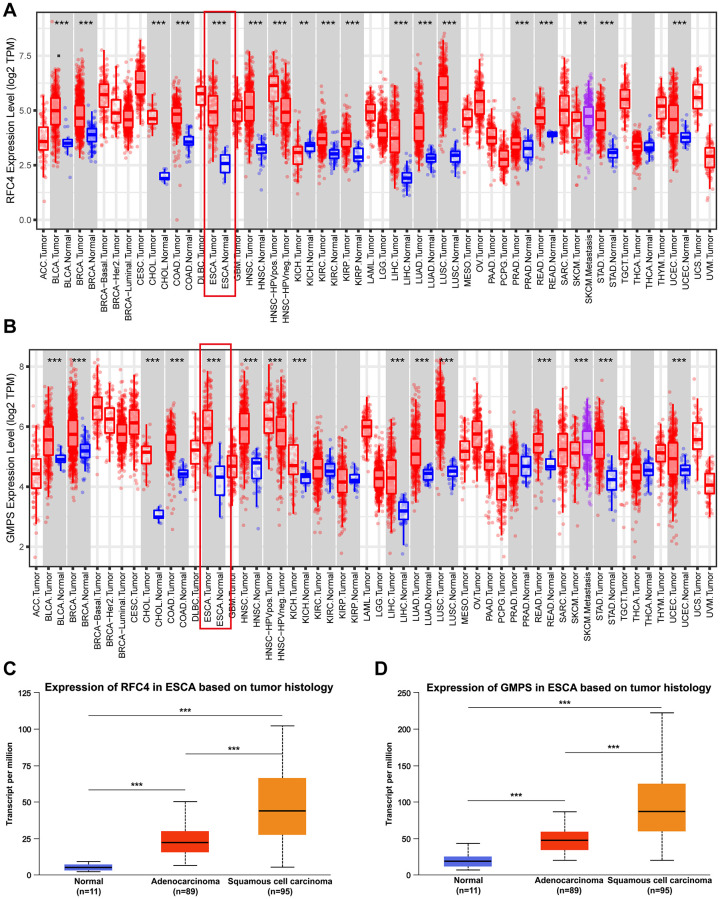
**RFC4 and GMPS were upregulated in most cancers, especially ESCC.** (**A**) Pan-cancer analysis of RFC4. (**B**) Pan-cancer analysis of GMPS. (**C**) Expression of RFC4 in patients with esophageal carcinoma based on histology. (**D**) Expression of GMPS in patients with esophageal carcinoma based on histology. ^*^p < 0.05, ^**^p < 0.01, ^***^p < 0.001.

By conducting an online TCGA analysis, we found that the expression of RFC4 and GMPS was upregulated in many cancers (Figure 4A, 4B). Then, we focus our attention on ESCA. By conducting a UALCAN online analysis, the results indicated that RFC4 and GMPS were increased significantly in esophageal carcinoma compared to normal tissues, especially in esophageal squamous cell carcinoma (Figure 4C, 4D).

**Figure 5:**
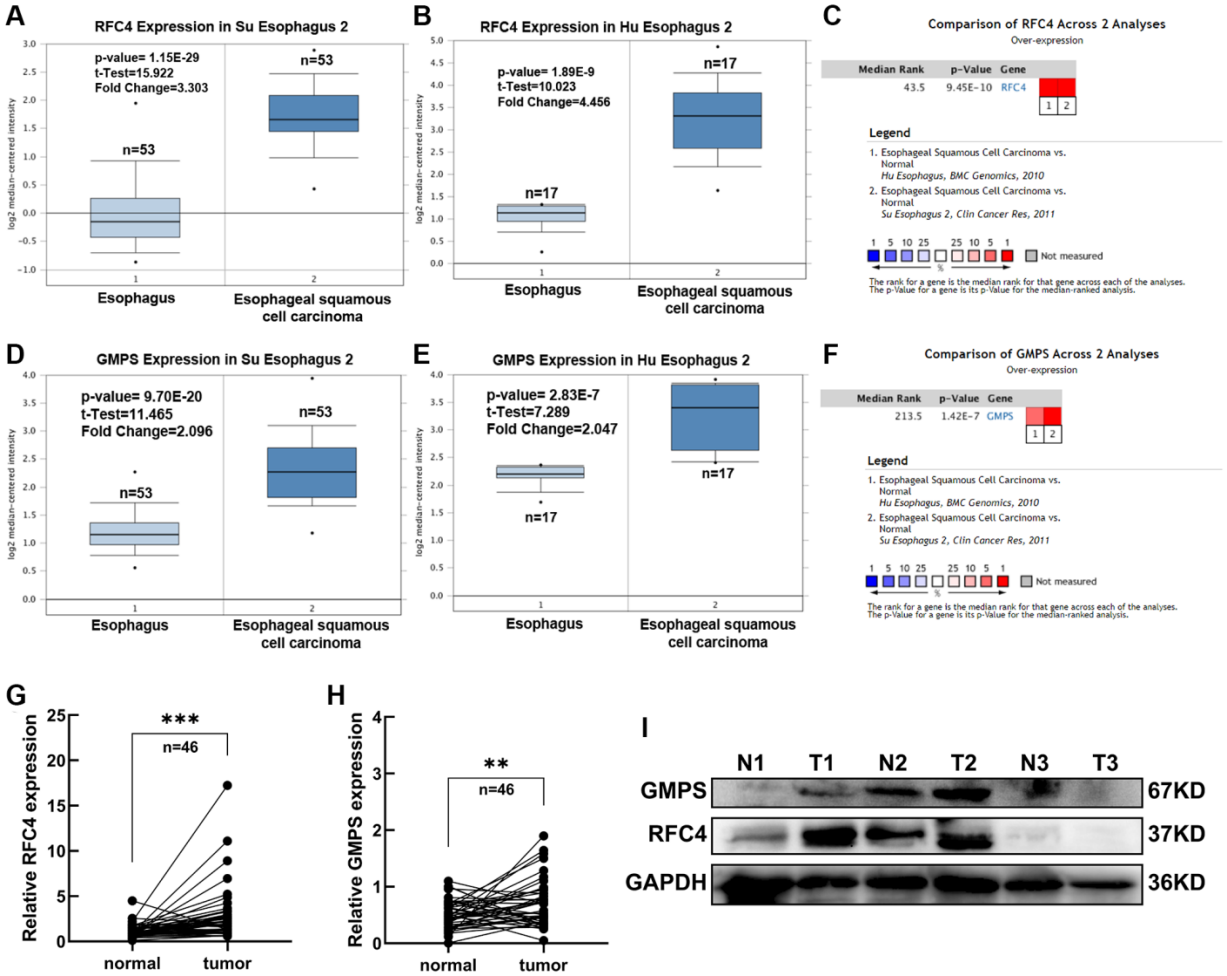
**Expression analysis of RFC4 and GMPS in ESCC.** (**A**) RFC4 mRNA levels in Su Esophagus 2. (**B**) RFC4 mRNA levels in Hu Esophagus 2. (**C**) Comparison of RFC4 across Su and Hu Esophagus. (**D**) GMPS mRNA levels in Su Esophagus 2. (**E**) GMPS mRNA levels in Hu Esophagus 2. (**F**) Comparison of GMPS across Su and Hu Esophagus. (**G**, **H**) RFC4 and GMPS mRNA levels were detected by RT-qPCR in 46 pairs of ESCC and adjacent normal tissues. (**I**) Protein levels in the three pairs of tissues. ^*^p < 0.05, ^**^p < 0.01, ^***^p < 0.001.

In order to further explore the expression of RFC4 and GMPS in the esophageal carcinoma, the Oncomine dataset was used. We found that RFC4 and GMPS were both increased in the two different ESCC datasets (Su and Hu Esophagus datasets) [17, 18] (Figure 5A–5F).

Therefore, we measured the expression levels of RFC4 and GMPS in 46 pairs of ESCC tumor samples and adjacent normal tissues to prove RFC4 and GMPS were increased in ESCC. Compared to the paired normal tissues, the results showed that RFC4 and GMPS were upregulated in the ESCC tumor tissues (Figure 5G, 5H). The protein levels of the two pairs of tissues were also significantly increased among the three pairs of tissues (Figure 5I).

### RFC4 and GMPS were upregulated in the early stage of esophageal carcinoma and may be biomarkers for the early diagnosis of esophageal carcinoma

**Figure 6:**
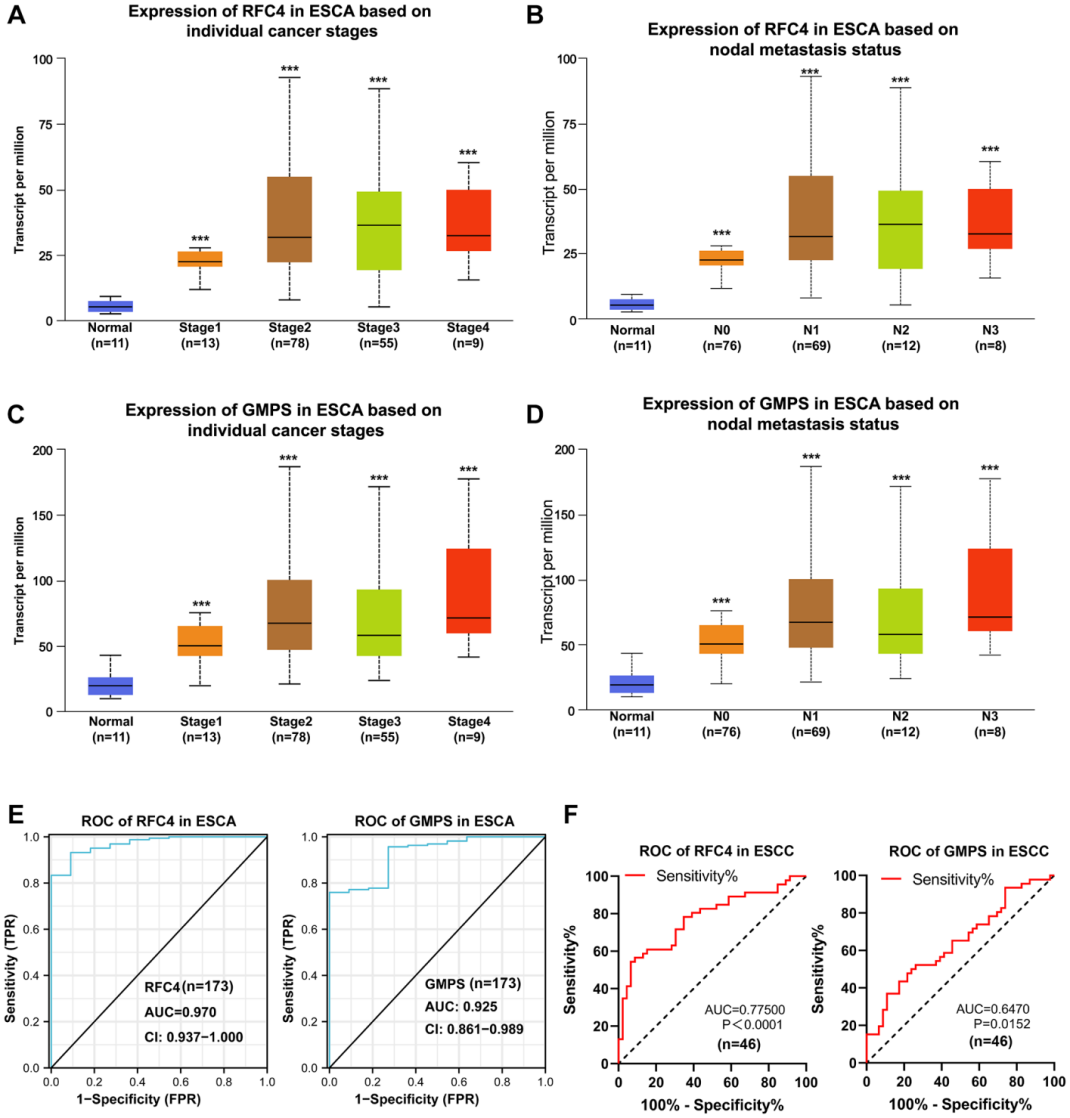
**Upregulated RFC4 and GMPS are associated with the early diagnosis of esophageal carcinoma and may be the biomarkers for the early diagnosis of esophageal carcinoma.** (**A**, **B**) Expression of RFC4 in patients with esophageal carcinoma based on the stage and nodal metastasis. (**C**, **D**) Expression of GMPS in patients with esophageal carcinoma based on the stage and nodal metastasis. (**E**) Receiver operating characteristic (ROC) curve analysis of RFC4 and GMPS in esophageal carcinoma (*n* = 173). (**F**) Receiver operating characteristic (ROC) curve analysis of RFC4 and GMPS in ESCC (*n* = 46). ^*^p < 0.05, ^**^p < 0.01, ^***^p < 0.001.

To found more evidences of RFC4 and GMPS participate in ESCC, the further analysis of UALCAN revealed that RFC4 and GMPS were significantly elevated in stage 1 esophageal carcinoma (Figure 6A, 6C). We also found that RFC4 and GMPS were upregulated at N0 based on the nodal metastasis status analyzed (Figure 6B, 6D). This funding may indicate that the expression of RFC4 and GMPS has increased, but metastasis did not occur yet or may be in the early stages. This funding was meaningful. Furthermore, combined with the ROC curves of 173 patients with ESCA and 46 pairs of ESCC samples (Figure 6E, 6F), we preliminarily concluded that RFC4 and GMPS are significant for identifying esophageal carcinoma in the early stage. In summary, we believe RFC4 and GMPS can be biomarkers for esophageal carcinoma identification and early diagnosis.

Moreover, to further investigate the association between the expression levels of RFC4 and GMPS and the clinicopathological features in ESCC, we divided the 46 tumor samples into two groups according to the cutoff values (median of RFC4, mean of GMPS) of RFC4 and GMPS mRNA expression levels. Next, we explored the correlation between RFC4 and GMPS expression and the clinicopathological parameters of patients with ESCC. A chi-square test was performed for the statistical analysis. Associations were observed between GMPS expression and vascular invasion (*p* = 0.017, Table 2). However, we did not find an association with RFC4 (Supplementary Table 2), but we did not have enough samples.

**Table 2:** Relationship between GMPS expression and clinicopathological features in ESCC.

**Clinical factor**	**Cases (*n* = 46)**	**GMPS expression**		***χ*^2^**	***p*-value**
		**Low (*n* = 24)**	**High (*n* = 22)**		
Gender					
Male	32	15	17	0.030	0.863
Female	14	9	5		
Age (years)					
<61	19	10	9	0.003	0.958
≥61	27	14	13		
BMI					
18.5–23.9	35	17	18	0.761	0.383
<18.5 OR ≥24	11	7	4		
Smoking status					
Yes	18	11	7	0.947	0.331
No	28	13	15		
Differentiation					
Well (G1)	4	2	2	0.170	1.000
Moderate (G2)	21	11	10		
Poor (G3)	21	11	10		
pT status					
Tis-2	12	5	7	0.947	0.331
T3-4	34	19	15		
pN status					
N0	32	16	16	0.199	0.665
N1-3	14	8	6		
Pathological stage					
0 + I + II	30	15	15	0.163	0.686
III + IV	16	9	7		
Vascular invasion					
Yes	5	1	4	5.690	0.017^*^
No	41	23	18		

### Upregulated RFC4 and GMPS levels may be mediated by increased DNA copy number in ESCC

**Figure 7:**
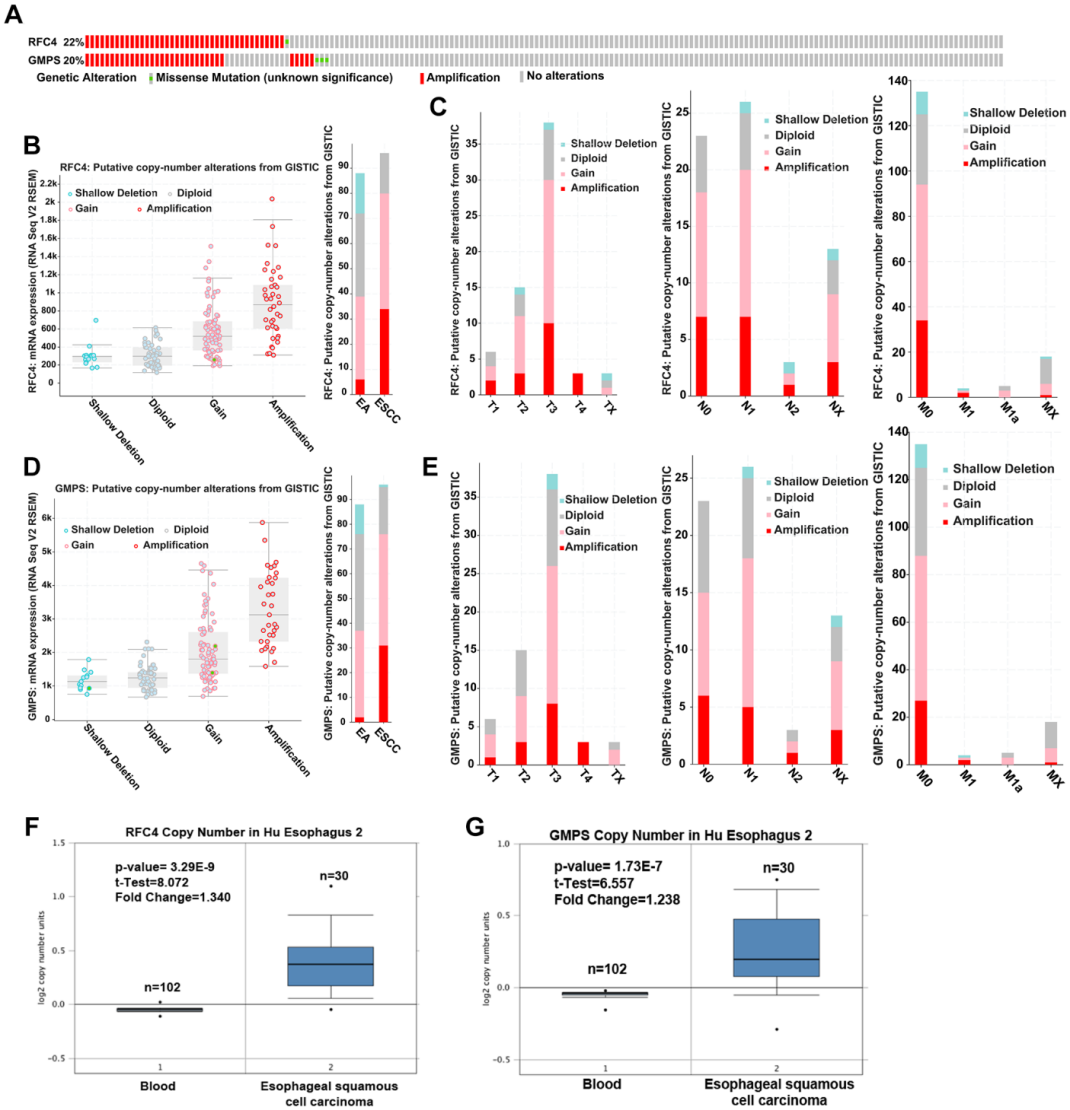
**Upregulated RFC4 and GMPS levels may be mediated by DNA copy number alterations in ESCC.** (**A**) Genetic alteration analysis of RFC4 and GMPS by cBioPortal. (**B**, **D**) Putative copy number alterations of RFC4 and GMPS in esophageal cancer. (**C**, **E**) Putative copy number alteration analysis of RFC4 and GMPS based on different T, N, and M stages. (**F**, **G**) DNA copy number of RFC4 and GMPS in Hu Esophagus 2. ^*^p < 0.05, ^**^p < 0.01, ^***^p < 0.001.

To further explore the reason for the increased RFC4 and GMPS in ESCC, we first explored genetic alterations in RFC4 and GMPS in esophageal carcinoma by a cBioPortal analysis. The results showed that amplification occurred in RFC4 and GMPS (RFC4, 40 cases (21.7%); GMPS, 33 cases (17.9%)) in 184 patients with esophageal cancer (Figure 7A). Further analysis found that gain and amplification were the most common copy number variations in esophageal carcinoma, especially ESCC (Figure 7B, 7D). Furthermore, for either RFC4 or GMPS, gain and amplification appeared in T1, N0 and M0. Therefore, we inferred that DNA copy number alterations mediated the increase in RFC4 and GMPS in the early stage of esophageal carcinoma (Figure 7C, 7E).

In addition, the DNA copy number was significantly increased in the ESCC patients based on an Oncomine online analysis (Figure 7F, 7G).

Some studies have reported that copy number changes may lead to cancer [19] and are associated with cancer patient prognosis [20]. Therefore, we inferred that the levels of RFC4 and GMPS may be mediated by an increased DNA copy number and related to the occurrence of esophageal cancer.

### Exploration of the mechanisms of RFC4 and GMPS by GSEA and correlation analysis

**Figure 8:**
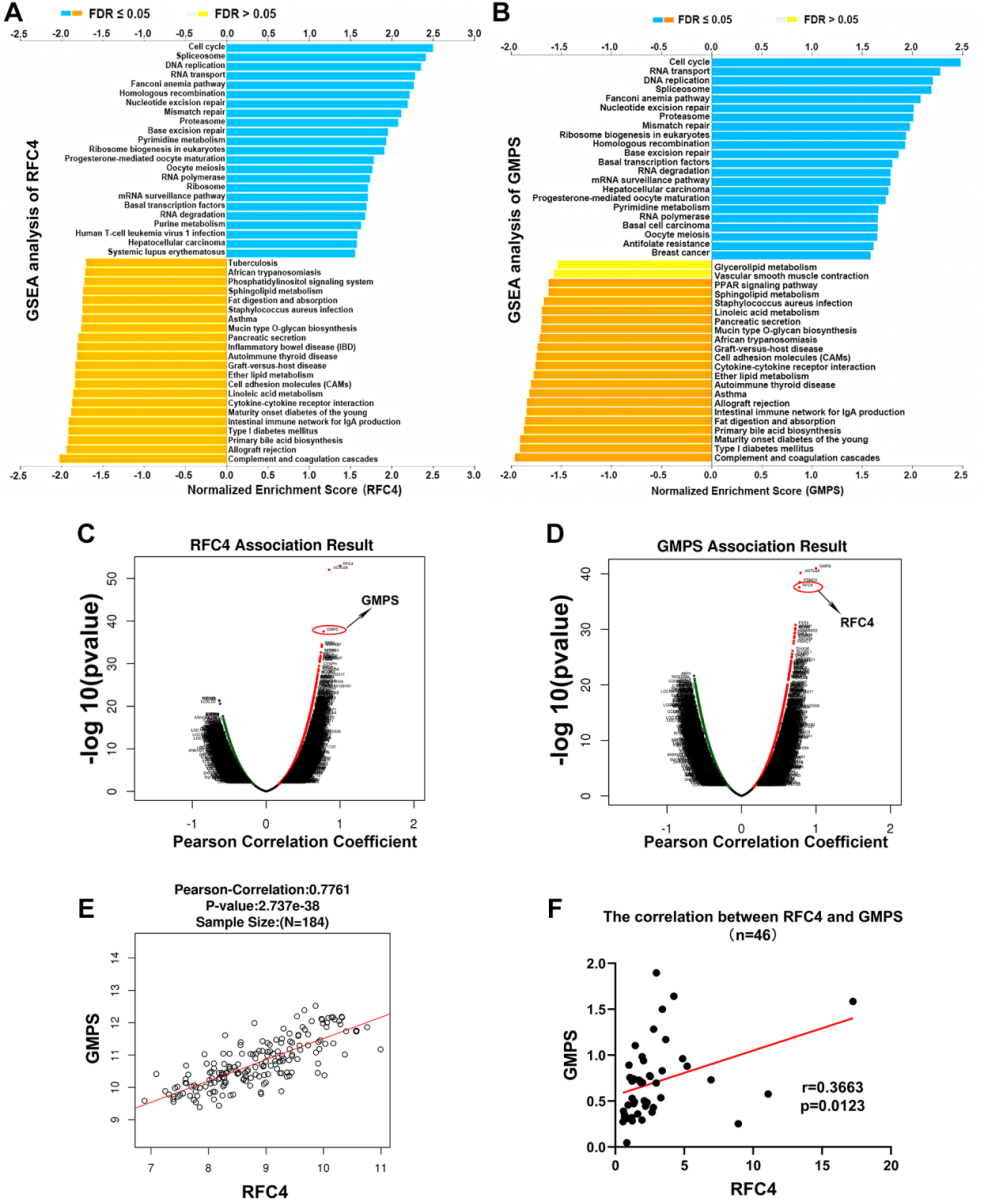
**Exploration of the mechanism of RFC4 and GMPS based on GSEA and correlation analysis.** (**A**, **B**) KEGG pathway analysis of RFC4 and GMPS based on GSEA. (**C**, **D**) Correlation coefficient analysis of RFC4 and GMPS in esophageal carcinoma. (**E**) Correlation between RFC4 and GMPS in TCGA. (**F**) Correlation between RFC4 and GMPS in 46 tumor samples. ^*^p < 0.05, ^**^p < 0.01, ^***^p < 0.001.

To explore the potential molecular mechanisms of RFC4 and GMPS in esophageal carcinoma, GSEA from LinkedOmics was used. The GSEA results indicated that the top 4 pathways of the high expression in the RFC4 group were the cell cycle, spliceosome, DNA replication and RNA transport pathways (Figure 8A). In the high GMPS group, the top 4 pathways were the cell cycle, RNA transport, DNA replication and spliceosome pathways (Figure 8B). Surprisingly, we found that the functional enrichment of the two genes highly overlapped. Therefore, we subsequently explored the relationship between RFC4 and GMPS. The results indicated that the expression of RFC4 was highly correlated with the expression of GMPS in esophageal carcinoma in TCGA data (Figure 8C–8E). This correlation was also confirmed in our 46 pairs of samples (Figure 8F). Based on these findings, we hypothesized that there might be a synergistic relationship between RFC4 and GMPS.

### RFC4 and GMPS expression is correlated with tumor-infiltrating immune cells and immune escape in esophageal carcinoma

**Figure 9:**
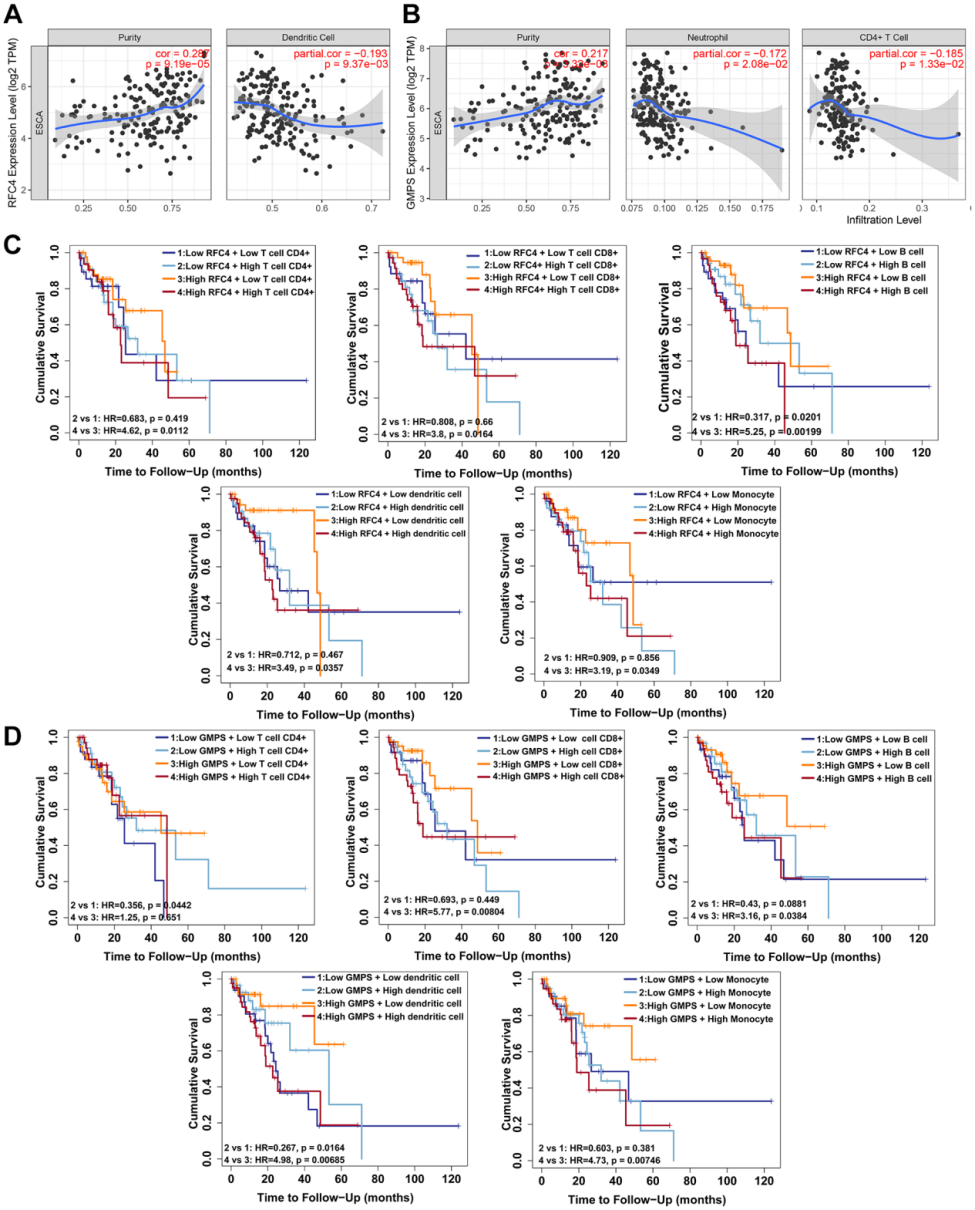
**RFC4 and GMPS expression correlates with tumor-infiltrating immune cells and immune escape in esophageal carcinoma.** (**A**) RFC4 expression was positively correlated with the tumor purity (r = 0.287, *p* < 0.05) but negatively correlated with Dendritic cells (r = −0.193, *p* < 0.05). (**B**) GMPS expression was positively correlated with the tumor purity (r = 0.217, *p* < 0.05) but negatively correlated with CD4+ T cells (r = −0.185, *p* < 0.05) and neutrophils (r =−0.172, *p* < 0.05). (**C**) A high level of RFC4 accompanied by a high expression of CD4+ T cells (*p* = 0.0112), CD 8+ T cells (*p* = 0.0164), B cells (*p* = 0.00199), dendritic cells (*p* = 0.0357) and monocytes (*p* = 0.0349) indicated a poor prognosis. When RFC4 was expressed at low levels, a high expression of B cells (*p* = 0.0201) was associated with a better prognosis. (**D**) A high level of GMPS accompanied by a high expression of CD 8+ T cells (*p* = 0.00804), B cells (*p* = 0.0384), dendritic cells (*p* = 0.00685) and monocytes (*p* = 0.00746) indicated a poor prognosis. When GMPS was expressed at low levels, a high expression of CD 8+ T cells (*p* = 0.0442) and dendritic cells (*p* = 0.0164) was associated with a better prognosis. *p* < 0.05 was considered significant.

In recent years, tumor-infiltrating immune cells have been shown to participate in tumor growth and tumor development [21, 22]. Therefore, we investigated the relationship between RFC4 and GMPS expression and immune infiltration in esophageal carcinoma using the online tool TIMER. Six immune cell types and the tumor purity were assessed by TIMER. The results showed a significant correlation between RFC4 expression and tumor purity and dendritic cells (Figure 9A). And the GMPS expression was related to tumor purity, CD4+ T cells and neutrophils (Figure 9B). Further analysis revealed that a high expression of RFC4 also indicates poor prognosis, even when accompanied by high levels of tumor-infiltrating immune cells, including CD4+ T cells, CD8+ T cells, B cells, dendritic cells and monocytes (Figure 9C). When RFC4 was expressed at low levels, the high expression of B cells was associated with a better prognosis (Figure 9C). Similarly, a high GMPS expression accompanied by high levels of CD4+ T cells, CD 8+ T cells, B cells, dendritic cells and monocytes also indicated a poor prognosis (Figure 9D). A low expression of GMPS with a high expression of CD4+ T cells and dendritic cells showed a good prognosis (Figure 9D). In summary, we speculate that RFC4 and GMPS overexpression might influence tumor immune responses in the tumor microenvironment and that they may play a crucial role in esophageal carcinoma progression.

## DISCUSSION

Esophageal carcinoma, especially ESCC, is a common health issue worldwide and usually has high mortality due to a late diagnosis and lack of efficient treatments [23, 24]. Usually, because the symptoms of the tumor are not specific during the early stages, endoscopy is used to screen early esophageal cancer [25, 26]. However, endoscopy has many limits. Endoscopy is expensive and not sufficiently available in many high-risk regions [23]. Therefore, a cheap, effective and acceptive diagnostic method is needed for early ESCC diagnosis.

In the present study, 3 datasets were analyzed to obtain DEGs between ESCC tissues and normal tissues. In total 64 DEGs were identified, including 22 downregulated genes and 42 upregulated genes. To gain more insight into ESCC, we found that RFC4 and GMPS in ESCC have rarely been investigated, and further studies are necessary.

RFC4 is a known subunit of the replication factor C complex, which functions mainly in DNA replication [27]. Many reports have shown that RFC4 may play an important role in the proliferation, progression, invasion, and metastasis of cancer cells [28]. Some findings suggest that RFC4 may be a potential prognostic biomarker and therapeutic target. For example, in colorectal cancer, RFC4 was correlated with tumor progression and predicted prognosis [29]. In another study, RFC4 decreased the growth and increased the chemosensitivity of hepatocellular carcinoma cells [30]. He et al. found that RFC4 was associated with significant survival in cervical squamous carcinoma [31]. In addition, RFC4 can act as a radio resistance factor in colorectal cancer [32]. However, few studies investigated ESCC. In our study, we found that RFC4 was increased in the early stage of esophageal carcinoma. By conducting a deep analysis, we inferred that DNA copy number alterations may mediate the elevation in genes.

GMPS catalyzes the final step in the de novo synthesis of guanine monophosphate [33]. It has been reported that GMPS plays a key role in cell proliferation and DNA replication. An early study also speculated that GMPS was a potential target for immunosuppressive therapy [34]. Recently, Zhang et al. found that TRIM21–SERPINB5 inhibits GMPS to protect nasopharyngeal carcinoma cells from radiation-induced apoptosis [35]. Wang et al. discovered that the inhibition of GMPS blocks prostate cancer growth [36]. Interestingly, our study is the first to report that GMPS is associated with ESCC. Similar to RFC4, GMPS was also increased in ESCC. In addition, the amplification of GMPS was also enhanced.

Altogether, we concluded that the increase in RCF4 and GMPS may be mediated by DNA copy number alterations, and that the increased expression of RFC4 and GMPS was associated with an early tumor stage and early nodal metastatic status in esophageal carcinoma. We inferred that RFC4 and GMPS can be biomarkers for esophageal carcinoma identification and early diagnosis. It was meaningful for early detection and diagnosis of esophageal cancer, and may improve the survival of patients with esophageal carcinoma. In addition, the GSEA showed that RFC4 and GMPS were significantly enriched in cell cycle, spliceosome, DNA replication and RNA transport. The function of RFC4 and GMPS in esophageal cancer were highly consistent. Through a correlation analysis, RFC4 was found to be strongly correlated with GMPS. We strongly considered that a synergistic relationship may exist between RFC4 and GMPS.

In recent years, immunotherapy has provided new hope for patients with cancers [37, 38]. Tumor-infiltrating immune cells are important for effective antitumor immunity [39, 40]. An early study showed that a high degree of CD8+ and CD4+ T-cell infiltration in ESCC was correlated with favorable clinical outcomes [41]. NK cells also play a vital role in ESCC [42]. In addition, our study explored the relationship between tumor-infiltrating immune cells and esophageal cancer. We uncovered that a high expression of RFC4 and GMPS accompanied by high levels of tumor-infiltrating immune cells was associated with a poor prognosis. However, a low expression of RFC4 and GMPS with a high expression of some tumor-infiltrating immune cells showed a good prognosis. Therefore, we reasonably concluded that RFC4 and GMPS involved in the immune regulation of esophageal cancer. A high expression of RFC4 and GMPS could mediate immune escape from esophageal cancer. In future, RFC4 and GMPS may serve as the targets for immunotherapy of esophageal cancer and improve the treatment of esophageal cancer.

However, the present study also has certain limitations, and further experiments need to be performed. Batch errors between many datasets cannot be avoided during analyses.

In conclusion, based on three datasets, we identified RFC4 and GMPS, which were upregulated in ESCC. Further analysis preliminarily revealed that RFC4 and GMPS are significant in the early stage and metastases and are possibly mediated by DNA copy number alterations. Additionally, GMPS was associated with vascular invasion in 46 tumor samples based on a clinical data analysis. In addition, RFC4 and GMPS perform the similar functions, and the expression of RFC4 was highly correlated with the expression of GMPS in esophageal cancer. Through a tumor-infiltrating immune analysis, we found that RFC4 and GMPS were correlated with some tumor-infiltrating immune cells and that an increased expression of RFC4 and GMPS could result in a poor prognosis. Finally, we concluded that RFC4 and GMPS are significant for the early diagnosis of esophageal carcinoma and that they may participate in the tumor immune response. Although the further experimental studies based on our findings are necessary, our findings are significant for the early diagnosis and treatment of ESCC.

## MATERIALS AND METHODS

### Data collection and identification of DEGs

GSE20347 [43] and GSE17351 [44] from GEO and our private sequencing data of ESCC were selected for our study. For the GEO datasets, the selection criteria were as follows: 1) tissues are diagnosed with ESCC and have matched normal tissues; and 2) probes can be converted into gene symbols and include complete information for the analysis. Finally, the three datasets contained 25 ESCC samples and 25 matched normal tissues.

The GEO2R tool (https://www.ncbi.nlm.nih.gov/geo/geo2r/) was applied to screen the DEGs between the tumor and nontumor samples. The DEGs were screened by |log_2_FC| > 1 and an adjusted *p-*value < 0.05. Then, online Venn diagram software (http://bioinformatics.psb.ugent.be/webtools/Venn/) was used for visualization of the data.

### PPI network construction and enrichment analysis of DEGs

First, we used the STRING (https://string-db.org/) database to construct protein-protein interaction (PPI) networks of the DEGs in ESCC. Then, we used Cytoscape software [45] to visualize the PPI networks.

The R package “clusterProfiler” was used for the GO enrichment and KEGG pathway analyses of the DEGs. *P <* 0.05 indicated statistical significance.

### Hub gene selection and analysis

The cytoHubba module of Cytoscape was used to screen hub genes based on a degree >4. Then, functional and pathway enrichment analyses of the hub genes were performed with the R package “clusterProfiler”.

### Expression analysis of RFC4 and GMPS in public datasets

The expression of RFC4 and GMPS in cancers was examined in the TIMER database [46, 47]. The mRNA expression and DNA copy number of RFC4 and GMPS in ESCC were further analyzed by using the Oncomine database (https://www.oncomine.org/resource/main.html). The relationship between the mRNA levels of RFC4/GMPS and the pathological features of patients with esophageal carcinoma in terms of the cancer stage and nodal metastasis was analyzed using UALCAN [48].

### Genetic alteration analysis using cBioPortal

cBioPortal (https://www.cbioportal.org/) is an open-access resource for multidimensional cancer genomic data [49, 50]. We used cBioPortal to explore genetic alterations in RFC4 and GMPS.

### Gene set enrichment analysis (GSEA) and correlation analysis using LinkedOmics

The GSEA of 184 esophageal carcinoma samples from TCGA was performed using LinkedOmics (http://www.linkedomics.org/admin.php datasets. The relationship of RFC4 and GMPS in ESCC was also analyzed by using LinkedOmics.

### Tumor-infiltrating immune cell (TILs) analysis

The online tool TIMER (https://cistrome.shinyapps.io/timer/) [46, 47] was used to investigate the correlation between RFC4/GMPS and the tumor purity and infiltrating immune cells (B cells, CD8+ T cells, CD4+ T cells, macrophages, neutrophils and dendritic cells). TIMER2.0 (http://timer.cistrome.org/) [51] was used for the survival analysis of RFC4 and GMPS with the tumor immune cell infiltration.

### Clinical samples

Forty-six pairs of fresh ESCC and adjacent normal tissues were collected from the Department of Thoracic Surgery at the First Affiliated Hospital of Sun Yat-Sen University (FAHSYSU). All tissue samples were directly frozen, and RNA was extracted in a timely manner. All tissue samples were endorsed by the Medical Ethical Committee of the SYSUCC and the FAHSYSU, and written consent documents were obtained from all patients.

### RNA extraction and quantitative real-time PCR analysis (qRT-PCR)

The total RNA was isolated by TRIzol reagent (Invitrogen). Reverse transcription and qRT-PCR were carried out using SYBR Green Master Mix (YEASEN) according to the manufacturer’s protocol. GAPDH was used as an internal control. The sequences of the primers were as follows:

GAPDH forward, 5′-GGAGCGAGATCCCTCCAAAAT-3′;
GAPDH reverse, 5′-GGCTGTTGTCATACTTCTCATGG-3′;
RFC4 forward, 5′-GGCAGCTTTAAGACGTACCATGG-3′;
RFC4 reverse, 5′-TCTGACAGAGGCTTGAAGCGGA-3′;
GMPS forward, 5′-CCCATCACAATGACACAGAGCTC-3′;
GMPS reverse, 5′-CTGGAAGTCCAAGTTCTCTGCC-3′.

The relative expression was calculated using the 2^−ΔΔCt^ method.

### Western blotting analysis

A Western blot analysis was performed according to standard methods [52]. A BCA Protein Quantification Kit (YEASEN) was used to measure the concentration. The antibodies against RFC4 and GMPS were purchased from Proteintech. GAPDH was used as an internal control.

### Statistical analysis

The data were analyzed using GraphPad Prism 8.0. The expression levels of the DEGs between the tumor and adjacent normal tissues were compared by paired two- tailed *t*-test. A chi-square test was performed to evaluate the relationship between the clinicopathological features and the expression levels of RFC4 and GMPS. A *p*-value <0.05 was considered statistically significant.

## Supplementary Materials

Supplementary Tables
