# Supplementary material for: The upregulated expression of RFC4 and GMPS mediated by DNA copy number alteration is associated with the early diagnosis and immune escape of ESCC based on a bioinformatic analysis
Source: Aging (Albany NY). 2021 Sep 14;13(17):21758–77. doi: 10.18632/aging.203520 (PMC8457608; doi:10.18632/aging.203520)
Supplement: Supplementary Tables [file aging-13-203520-s001.pdf]

SUPPLEMENTARY TABLES

Supplementary Table 1. The total of 64 DGEs.

Category	Genes
Downregulated genes	CRISP3, SPINK5, MAL, CYP4B1, PPP1R3C, CYP3A5, BBOX1, C2orf54, CFD, HPGD, GPX3, EMP1, FMO2, HSPB8, NUCB2, ABLIM1, RAB11A, SASH1, UBL3, CAST, MGST2, RIOK3
Up-regulated genes	YEATS2, ATP1B3, ATP2B1, GPNMB, PLOD1, PTDSS1, ATP2C1, U2SURP, RFC4, RSRC1, GMPS, PRKDC, CKS1B, TFRC, ARPC1B, FZD6, PAK2, HLTF, APMAP, ITGA6, TOP2A, COL7A1, ECT2, MCM2, MEST, CENPF, ITGB4, LAMB3, BGN, CDH3, ENAH, SERPINH1, PLAU, LAMC2, MMP10, APOC1, COL10A1, MMP11, ADAM12, NELL2, COL11A1, MMP13

Supplementary Table 2. Relationship between RFC4 expression and clinicopathological features in ESCC.

Clinical factor	Cases (*n* = 46)	RFC4 expression		χ^2	*p*-value
		Low (*n* = 23)	High (*n* = 23)		
Gender					
Male	32	15	17	0.411	0.522
Female	14	8	6		
Age (years)					
<61	19	11	8	0.807	0.365
≥61	27	12	15		
BMI					
18.5–23.9	36	17	19	0.511	0.475
<18.5 OR ≥24	10	6	4		
Smoking status					
Yes	25	10	15	2.190	0.139
No	21	13	8		
Differentiation					
Well (G1)	4	1	3	1.062	0.693
Moderate (G2)	21	11	10		
Poor (G3)	21	11	10		
pT status					
Tis-2	12	6	6	0.000	1.000
T3-4	34	17	17		
pN status					
N0	32	15	17	0.411	0.522
N1–3	14	8	6		
Pathological stage					
0 + I + II	30	14	16	0.383	0.536
III + IV	16	9	7		
Vascular invasion					
Yes	41	1	4	2.020	0.155
No	5	22	19		
